# Low-protein diet applied as part of combination therapy or stand-alone normalizes lifespan and tumor proliferation in a model of intestinal cancer

**DOI:** 10.18632/aging.203692

**Published:** 2021-11-12

**Authors:** Alina Proske, Judith Bossen, Jakob von Frieling, Thomas Roeder

**Affiliations:** 1 Department of Molecular Physiology, Zoological Institute, Kiel University, Kiel, Germany; 2 Airway Research Center North (ARCN), German Center for Lung Research (DZL), Kiel, Germany

**Keywords:** dietary protein restriction, afatinib, EGFR, *Drosophila*, stem cells, cancer

## Abstract

Tumors of the intestinal tract are among the most common tumor diseases in humans, but, like many other tumor entities, show an unsatisfactory prognosis with a need for effective therapies. To test whether nutritional interventions and a combination with a targeted therapy can effectively cure these cancers, we used the fruit fly *Drosophila* as a model. In this system, we induced tumors by EGFR overexpression in intestinal stem cells. Limiting the amount of protein in the diet restored life span to that of control animals. In combination with a specific EGFR inhibitor, all major tumor-associated phenotypes could be rescued. This form of treatment was also successful in a real treatment scenario, which means when they started after the full tumor phenotype was expressed. In conclusion, reduced protein administration can be a very promising form of adjuvant cancer therapy.

## INTRODUCTION

Gastrointestinal (GI) cancers account for about one-fourth of the world’s cancers as well as more than one-third of all cancer-associated deaths [1, 2]. Although there have been substantial advances in the treatment of most forms of GI cancers, the prognosis for most patients remains unacceptably poor. This situation is unlikely to improve in the coming years for most GI cancers, including colorectal cancer (CRC). This negative outlook is based on the premise that risk factors important for the development of CRC, such as cigarette and alcohol consumption and various forms of obesity, continue to increase [3]. These factors, which promote cancer, also include certain food-consumption patterns, including the so-called Western diet, which is particularly rich in fat and carbohydrates [4]. Epidemiological studies show that specific dietary habits significantly reduce the risk of developing cancer [4, 5]. This finding that different nutritional regimens have a dramatic impact on tumor incidence has caused research to focus on the specific nutritive requirements of tumor cells. Cancer cells require high levels of glucose and growth signals to survive and proliferate, making them particularly vulnerable to nutritional interventions [6]. Consequently, a diet that interferes with the specific metabolic requirements of cancer cells should inhibit tumor growth. One example of such a strategy is interfering with tumor glycolysis; an approach that, for various reasons, is rarely incorporated into therapy [7]. By contrast, another therapeutically relevant form of nutritional intervention, the use of dietary restriction (DR) or caloric restriction (CR), is more promising; DR or CR substantially increases healthspan and lifespan in many model organisms [8–11].

Two types of DR have highlighted the importance of dietary interventions in cancer therapy: a reduction in the amount of protein in the diet and time-restriction of food intake. For example, protein reduction in the diet leads to less proliferation in several tumor types [8, 9, 12]. In addition, a diet that mimics fasting has been successfully used as a supportive treatment in combination with conventional cancer therapy [13, 14]. Protein restriction can be achieved in several ways: by reducing the amount of single essential amino acid, by reducing the levels of specific non-essential amino acids, or by reducing the levels of all amino acids [15]. Reducing specific non-essential amino acids may reduce cellular proliferation, but cancer cells may adapt accordingly [16, 17]. A global reduction in amino acid supply results in two major outcomes: 1) a greatly reduced energy intake and 2) a reprogramming of cellular metabolism [15, 17]. The first outcome might be the reason why therapeutic approaches using this approach have so far remained largely unrealized in clinical practice [18]. Reduced protein intake, on superficial inspection, leads to a condition that is similar to tumor-associated cachexia, which is a common complication in cancer patients that often leads to death [19]. Tumor-associated cachexia is often associated with severe hypermetabolism leading to a negative energy balance and consequently to weight loss mostly affecting muscle mass [19]. Therefore, the energy intake requirement in these patients is increased, which is usually met by energy-rich diets [20, 21]. The lower energy intake that is conferred by DR could, in principle, favor the development of cachexia. However, DR could also counteract the development of cachexia by restoring normal metabolic processes and the corresponding signaling systems that are relevant for metabolic homeostasis [10, 22, 23]. Therefore, the positive influences of DR can far outweigh the negative ones. For this reason, research assessing the potential of dietary interventions in cancer therapy and a better understanding of the underlying processes is urgently needed.

In CRC, like in most other cancers, specific mutations in oncogenes drive tumor progression. Among the most relevant oncogenes in CRC is the epidermal growth factor receptor (EGFR). Together with its downstream targets such as Ras, Raf, or PI3K/Akt, EGFR plays a decisive role in the initiation and development of CRC [24–26]. Moreover, *EGFR* is overexpressed in 35–49% of all CRCs [27–29], with overexpression levels of between 25% and 82% [30]. EGFR is highly expressed in primary cell cultures of human colorectal carcinomas [31]. As a result, EGFR and downstream signaling molecules are promising targets for directed therapy in CRC.

Animal models that reflect the genetic situation in common human cancers have substantially increased our understanding of how a specific mutation causes tumor development. Besides murine models, the fruit fly *Drosophila melanogaster* is one of the most important cancer models. As well as enabling quick analysis and a wealth of available genetic tools, *Drosophila* cancer models allow therapeutic interventions to be assessed not only in terms of the tumor growth but also in terms of lifespan. The ability to measure lifespan — an important benchmark in cancer research — gives *Drosophila* models a unique advantage over vertebrate models. Importantly, *Drosophila* is an exceptional model for investigating intestinal cancers, since the majority of the highly conserved mutations associated with human intestinal cancer induce over-proliferation of *Drosophila* intestinal stem cells (ISCs) and lead to tumor formation [32–34]. Moreover, this system not only reproduces tumor formation but also recaptures cancer-associated phenotypes such as tumor-induced wasting of host organs [35].

In this study, we used *Drosophila melanogaster* to study the effects of DR (namely, protein restriction), an oncogene-specific pharmacological intervention and DR combined with the pharmacological intervention on tumor development and organismal survival in a stem cell-derived tumor model induced by overexpression of a constitutively active form of EGFR (*Egfr^CA^*). We showed that protein restriction combined with the EGFR inhibitor afatinib reduced tumor growth and normalized life span. By applying these interventions to established tumors, we demonstrated the long-term effectiveness of these interventions.

## RESULTS

The experiments in this study used expression of a constitutively active Egfr allele (*Egfr^CA^*) targeted to ISCs and enteroblasts (esg+ cells) via the binary Gal4/UAS expression system [36]. The TARGET (temporal and regional gene expression targeting) system was used to restrict expression to adults [37]. In this system, concomitant expression of a temperature-sensitive version of the Gal4 repressor Gal80 (Gal80^ts^) allows ectopic *Egfr^CA^* expression to be induced by increasing the temperature from 18°C (restrictive) to 29°C (permissive). Crossing to *w^1118^* served as control. Upon induction, treatment was immediately applied (later referred to as early application).

### DR reduces ISC over-proliferation and normalizes lifespan

**Figure 1:**
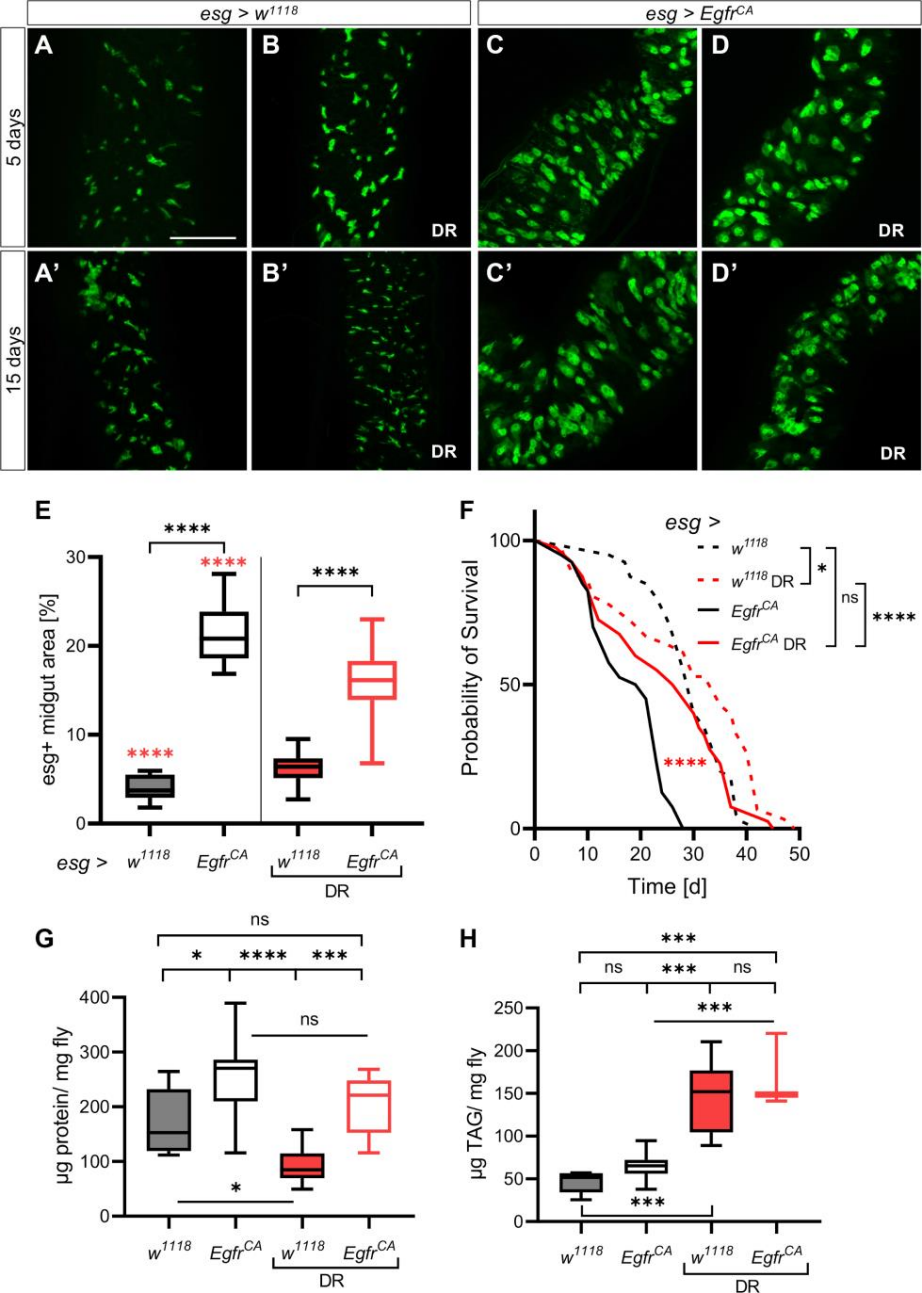
**Dietary restriction reduces the *Egfr^CA^*-induced phenotype.** Control animals (*esg > w^1118^*) and animals with *Egfr*-induced over-proliferation (*esg > Egfr^CA^*) in intestinal stem cells and enteroblasts (esg+ cells) were exposed to dietary restriction (DR) during induction. The esg+ cells are marked with GFP. (**A**, **B**) Intestines of control flies with and without exposure to DR after 5 days and 15 days (**A’**, **B’**). (**C**, **D**) Intestines of *Egfr^CA^* animals with and without exposure to DR after 5 days and 15 days (**C’**, **D’**). (**E**) Quantification of the area covered by GFP-positive cells indicating the number of esg+ cells in the midgut after 5 days of intervention. *n* = 10–13. (**F**) The lifespan of animals exposed to DR. *n* = 32–40. (**G**) Quantification of protein per mg fly after 5 days. *n* = 10–11. (**H**) Quantification of triacylglyceride (TAG) per mg fly after 5 days. *n* = 10. Statistical significance was tested by one-way ANOVA and the Tukey test. The lifespan significance was tested by the log-rank (Mantel-Cox) test. Significances are marked with lines or the corresponding color. ns = not significant, ^*^ = *p* < 0.05, ^***^ = *p* < 0.001, ^****^ = *p* < 0.0001. Scale bar: 100 μm.

A reduced dietary protein intake (that is, DR) inhibits tumor growth in several tumor models and generally increases lifespan in several types of organisms [9, 10, 12, 38, 39]. We investigated the effects of DR on the over-proliferation phenotype and lifespan in our *Drosophila* model. Animals were fed a modified holidic diet [40] and the ratio of protein to carbohydrate was reduced to 1:16. The protein to carbohydrate ratio was 1:1 in the control diet. The esg+ cells in the midgut were observed after 5 days and 15 days. There was no difference in the number and shape of esg+ cells in animals on a control diet (5 days old, Figure 1A; 15 days old, Figure 1A’) and animals on DR (5 days old, Figure 1B; 15 days old, Figure 1B’) under control conditions (no induction of the cancer phenotype). Expression of *Egfr^CA^* in esg+ cells caused over-proliferation and cell dysplasia in intestines (Figure 1C, C’) characterized by a visible increase in the number of GFP-positive cells with an abnormal phenotype compared with *w^1118^* controls (that is, cells without *Egfr^CA^* expression; Figure 1A) that were healthy. The abnormal phenotype was observed in flies that were 5 days old and in flies that were 15 days old (Figure 1C, 1C’). DR only marginally altered the over-proliferation phenotype (Figure 1D, 1D’). Nevertheless, there was a significantly lower number of esg+ cells in *Egfr^CA^* flies on DR than in *Egfr^CA^* animals fed a normal diet, confirming that DR alters the over-proliferation phenotype (Figure 1E). Transverse cuts through the abdomen showed that control guts have an epithelial monolayer while those of *Egfr^CA^* animals developed an epithelial bilayer with increased numbers and size of esg+ cells upon induction. DR was able to reinstate the epithelial monolayer but only marginally reduced esg+ cell size and number (Supplementary Figure 1). The lifespan of flies expressing *Egfr^CA^* was significantly lengthened upon DR and was similar to the lifespan of the *w^1118^* control fed a normal diet (Figure 1F). The median lifespan was only slightly reduced to 90%, while the maximum lifespan exceeded controls fed a normal diet (median, 27 d; maximum, 44 d, Table 1). DR increased the median lifespan of *w^1118^* significantly (110%, 33 d; maximum, 44.5 d, Figure 1F, Table 1). To exclude the possibility that DR induced a wasting phenotype, we measured the body composition of protein and fat (Figure 1G, 1H). Animals expressing *Egfr^CA^* had a higher body protein content than *w^1118^* controls, regardless of whether animals received a normal diet or underwent DR (Figure 1G, Supplementary Figure 2A). DR similarly increased the body fat content in both *w^1118^* control and *Egfr^CA^* animals (Figure 1H, Supplementary Figure 2B). This result indicates that DR does not induce significant wasting of the animals. Accordingly, DR reduced the number of esg+ cells and prolonged lifespan of *Egfr^CA^* animals to a wildtype level.

**Table 1:** Summarized median lifespans of applied interventions and situations.

**Group**	**Intervention**	**Lifespan**		
		**Median days**	**%**	**Maximum days**
*esg > w^1118^*	−	30	100	38.5
	DR (early)	33	110	44.5
*esg > Egfr^CA^*	−	20	66	28
	DR (early)	27	90	44
	100 μM Afatinib (early)	25	83	32
	50 μM Afatinib (early)	21	70	27
	Combination (100 μM) (early)	31	103	40
	Combination (50 μM) (early)	28	93	39.5
	Afatinib (late)	23	77	28
	DR (late)	33	110	38
	Combination (100 μM) (late)	28	93	38

DR = dietary restriction; early = treatment from onset on; late = treatment from day 5 on; maximum lifespan: mean survival of the oldest 10%.

### Afatinib reduces ISC over-proliferation and prolongs lifespan

**Figure 2:**
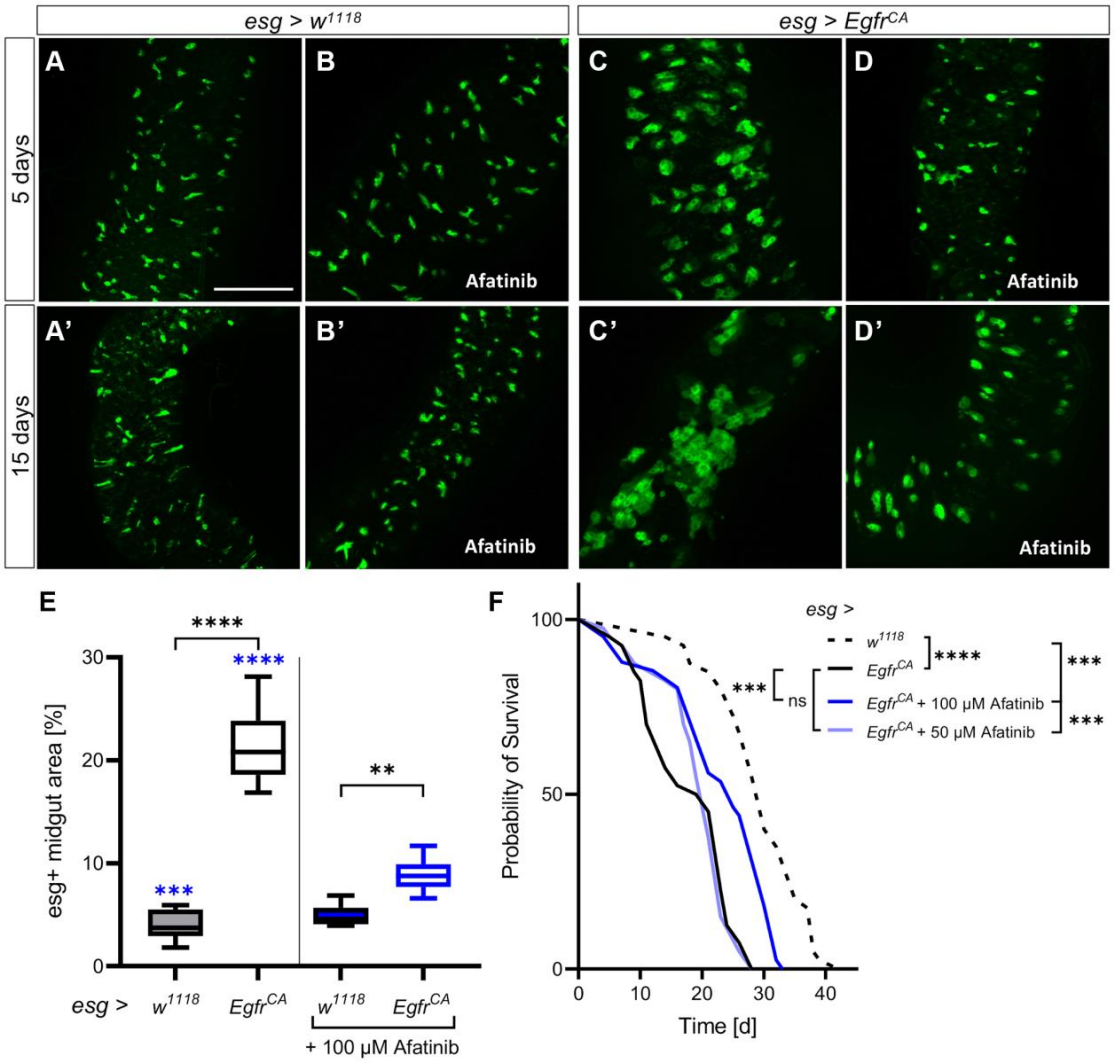
**Afatinib reduces the *Egfr^CA^*-induced phenotype.** Control animals (*esg > w^1118^*) and animals with *Egfr*-induced over-proliferation (*esg > Egfr^CA^*) of intestinal stem cells and enteroblasts (esg+ cells) were treated with afatinib (100 μM) during induction. The esg+ cells are marked with GFP. (**A**, **B**) Intestines of control flies with and without treatment after 5 days and (**A’**, **B’**) 15 days. (**C**, **D**) Intestines of *Egfr^CA^* animals with and without treatment after 5 days and (**C’**, **D’**) 15 days. (**E**) Quantification of the area covered by GFP-positive cells in midguts indicating the number of esg+ cells after 5 days of intervention. *n* = 10–13. (**F**) The lifespan of animals treated with 100 μM or 50 μM afatinib. *n* = 40. Statistical significance was tested by one-way ANOVA and the Tukey test. Lifespan significance was tested by the log-rank (Mantel-Cox) test. Significances are marked with lines or corresponding color. ns = not significant, ^**^ = *p* < 0.01, ^***^ = *p* < 0.001, ^****^ = *p* < 0.0001. Scale bar: 100 μm.

To assess the impact of a pharmacological intervention, we treated the animals with a specific EGFR inhibitor. We chose the second-generation EGFR inhibitor afatinib (BIBW2992), which is known to rescue a lethal *Egfr^CA^*-induced tumor phenotype in *Drosophila* trachea [41]. Afatinib is an approved treatment for EGFR-positive lung cancers, but it is not yet approved for the treatment of CRC. Microscopic analyses of GFP expression in esg+ cells of the midgut showed that there was no obvious difference in GFP expression between control animals without treatment (5 days old, Figure 2A; 15 days old, Figure 2A’) and those treated with 100 μM afatinib (5 days old, Figure 2B; 15 days old, Figure 2B’).

In animals with *Egfr^CA^* expression (Figure 2C, 2C’), afatinib reduced the number of GFP-positive cells to a similar number to those seen in *w^1118^* controls at 5 days (Figure 2D) and 15 days (Figure 2D’). This was also seen in the transverse cuts, were afatinib was able to reduce the number and size of esg+ cells (Supplementary Figure 1). Quantification of the area covered by GFP-positive cells confirmed that afatinib significantly reduced the number of esg+ cells at 5 days (Figure 2E). The lifespan of animals treated with afatinib was significantly longer (median, 25 d; maximum, 32 d) than the lifespan of animals that did not receive afatinib (Figure 2F, Table 1; median, 20 d; maximum, 28 d), and *w^1118^* controls (Figure 2F, Table 1; median, 30 d; maximum, 38.5 d). The median lifespan of afatinib-treated animals was 83% of that of the *w^1118^* control, which had a median lifespan of 30 days. In the above experiments, 100 μM afatinib was used; a lower concentration of afatinib (50 μM) did not rescue the reduced lifespan phenotype of *Egfr^CA^*-expressing animals (Figure 2F, Table 1). Thus, afatinib reduced the proliferation phenotype and prolonged the lifespan of *Egfr^CA^* animals when used at a concentration of 100 μM.

### Additional effects of DR and afatinib on Egfr-induced over-proliferation

**Figure 3:**
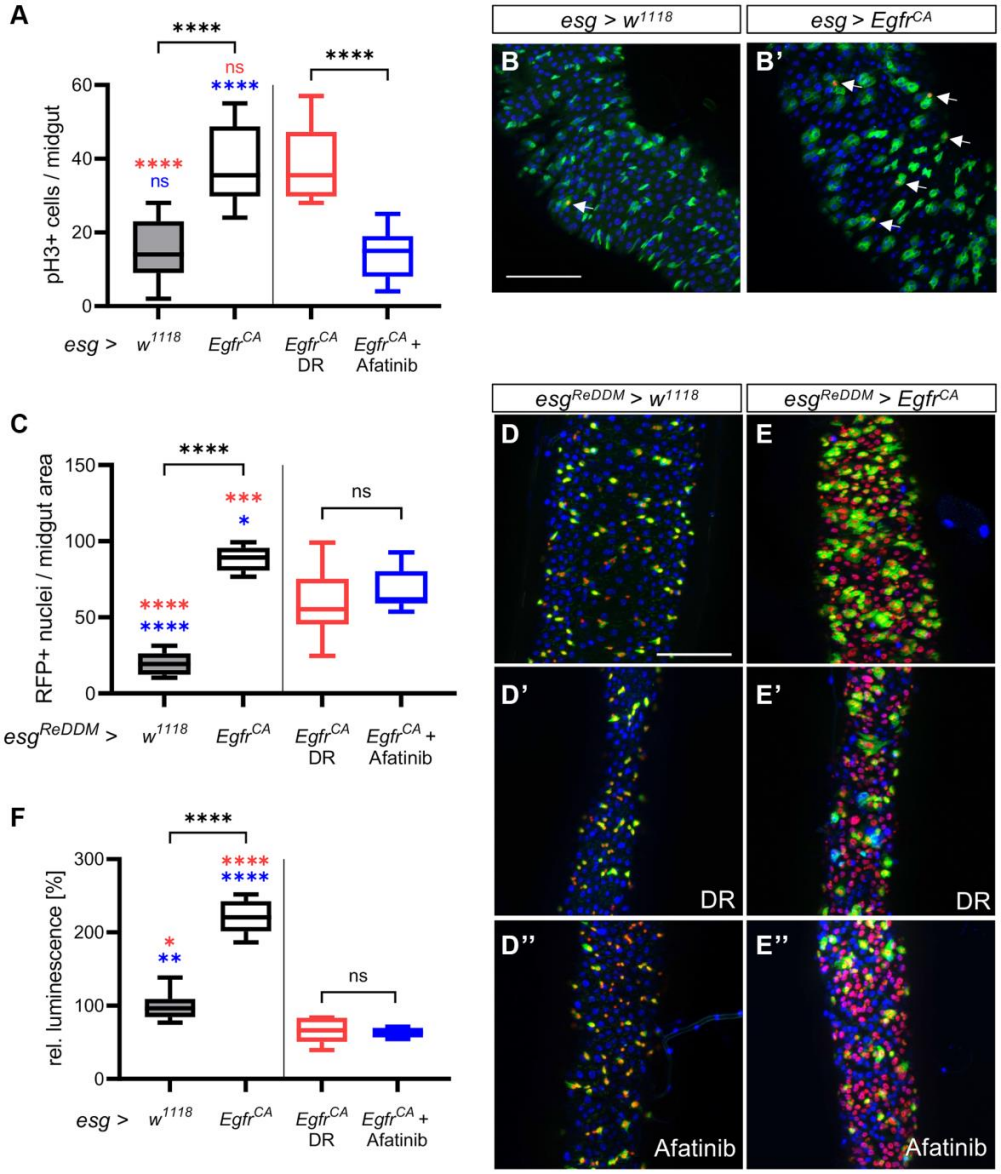
**Detailed phenotypic analysis of the effects of DR and afatinib.** Control animals (*esg > w^1118^*) and animals with an *Egfr*-induced over-proliferation (*esg > Egfr^CA^*) of intestinal stem cells and enteroblasts (esg+ cells) were either exposed to dietary restriction (DR) or treated with afatinib at induction. (**A**) Midguts were stained with an antibody for phospho-histone 3 to mark cells undergoing mitosis after 5 days. Ph3-positive stained cells in the whole intestine were counted. *n* = 9–11. (**B**, **B’**) Midguts of control animals (*esg > w^1118^*) and animals with *Egfr*-induced over-proliferation (*esg > Egfr^CA^*); esg+ cells are marked with GFP and mitotically active cells are stained red with an anti-pH3 antibody (indicated by the white arrows). (**C**) Quantification of cells that are RFP-positive through induction of the ReDDM system after 5 days. *n* = 10–11. (**D**–**E**) Control animals (*esg^ReDDM^ > w^1118^*) and animals with an *Egfr*-induced over-proliferation (*esg^ReDDM^ > Egfr^CA^*) in esg+ cells of the midgut were either exposed to DR at induction (**D’**, **E’**) or treated with afatinib (**D”**, **E”**). Esg+ cells are shown in green, RFP-positive progeny are shown in red, and nuclei are shown with blue DAPI staining. (**F**) Luciferase was quantified in whole animals as a measure of the over-proliferation phenotype after 15 days of induction. *n* = 5–7. Statistical significance was tested by one-way ANOVA and the Tukey test. Significances are marked with lines or the corresponding color. ns = not significant, ^*^ = *p* < 0.05, ^**^ = *p* < 0.01, ^***^ = *p* < 0.001, ^****^ = *p* < 0.0001. Scale bar: 100 μm.

We next investigated if DR and afatinib reduce the proliferation rate and mitotic activity of the stem cells. To measure the proliferation rate, we stained the intestines with an antibody for phospho-histone 3 (pH3) to mark mitotically active cells. The number of mitotically active stem cells was higher in the midguts of *Egfr^CA^* animals than in *w^1118^* controls (Figure 3A, 3B, 3B’). There was no difference in the number of pH3-positive cells in *Egfr^CA^* animals on DR compared with *Egfr^CA^* animals on a normal diet (Figure 3A). Afatinib reduced the proliferation of pH3-positive cells with *Egfr^CA^* expression to the level observed in *w^1118^* controls (Figure 3A). Furthermore, we used the ReDDM system (repressible dual differential marker), which marks cell components with fluorophores of varying stability, to investigate the cell turnover [42]. Here, we used *esg^ReDDM^* to mark esg+ cells with GFP and RFP. Since GFP has a shorter half-life than RFP, it is exclusively located in the cytoplasm of esg+ progenitor cells like ISC and enteroblasts. Due to its longer half-life, nucleus-located RFP is visible in all nuclei that develop after induction. When ReDDM is used together with the GAL4-UAS [36] and TARGET systems [37], cell turnover can be visualized in a temporal and tissue-specific manner. We quantified the cell turnover by quantifying the amount of RFP-positive cell progeny. The number of RFP+ nuclei in the midguts of *Egfr^CA^* animals exposed to DR and afatinib was significantly lower than in untreated *Egfr^CA^* animals fed a normal diet (Figure 3C). DR reduced the number of RFP+ nuclei to a greater extent (Figure 3D’, 3E’) than afatinib (Figure 3D”, 3E”) compared with the *Egfr^CA^* animals without intervention (Figure 3D, 3E). This effect was evident from microscopic images and quantification of these images (Figure 3C–3E). Luciferase expression was used to measure the increase in cell mass in the whole animal after 15 days of intervention. Luciferase activity was higher in the animals with *Egfr^CA^* expression in esg+ cells than in controls; this result was in agreement with the phenotype that we observed after quantification of GFP-positive esg+ cells.

In animals exposed to DR or treated with afatinib, luciferase expression was lower in *Egfr^CA^*-expressing animals than in *w^1118^* controls (Figure 3F). In *w^1118^* controls, DR significantly reduced luminescence, but 100 μM afatinib did not alter luminescence (Supplementary Figure 3). In conclusion, afatinib, but not DR, reverses the *Egfr*-induced increase in mitotic activity of the stem cells. DR reduces the cell turnover rate more effectively than afatinib.

### DR combined with afatinib restores the wild-type phenotype

**Figure 4:**
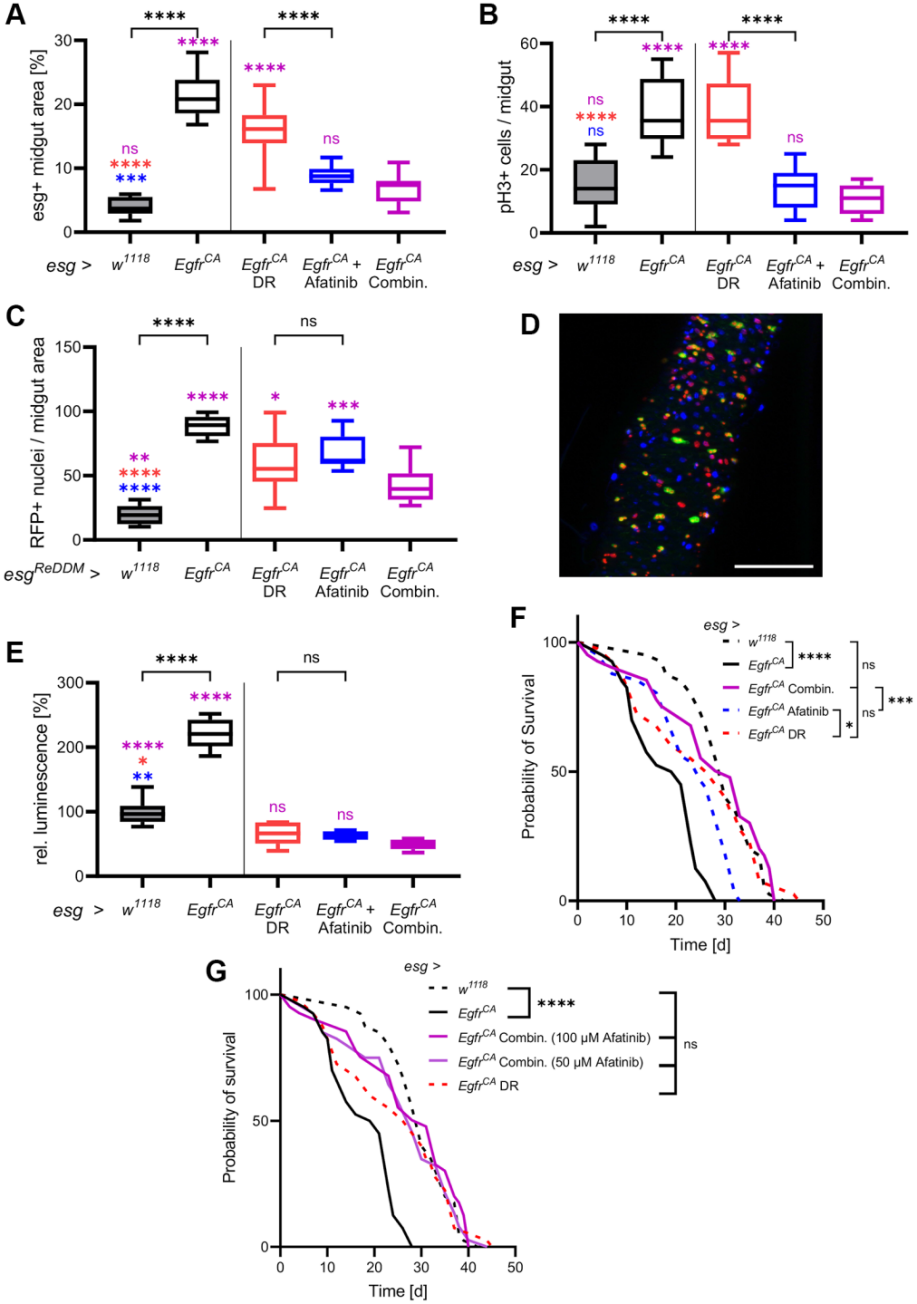
**DR combined with afatinib reduces cell proliferation and restores lifespan.** Control animals (*esg > w^1118^*) and animals with *Egfr*-induced over-proliferation (*esg > Egfr^CA^*) in intestinal stem cells and enteroblasts (esg+ cells) were exposed to a combination of DR and afatinib at induction (shown in magenta). Data were compared to animals that were exposed to dietary restriction (DR, red) or treated with afatinib (blue, 100 μM). (**A**) Quantification of the area covered by GFP-positive cells indicating the number and size of esg+ cells in the midgut after 5 days of induction. *n* = 10–13. (**B**) Midguts were stained with an antibody for phospho-histone 3 to mark cells undergoing mitosis after 5 days of induction. Positively stained cells in the whole midgut were counted. *n* = 9–11. (**C**) Quantification of cells that are RFP-positive after 5 days of induction of the ReDDM system. *n* = 10–11. (**D**) The combination of both treatments was analysed using the ReDDM system. Esg+ cells are shown in green, RFP-positive progeny are shown in red, and nuclei are shown with blue DAPI staining. (**E**) Luciferase and GFP were expressed simultaneously and luciferase activity was quantified in whole animals after 15 days of induction. *n* = 5–7. (**F**) The lifespan of animals exposed to DR, afatinib, or a combination of DR and 100 μM afatinib. *n* = 32–40. (**G**) The lifespan of animals exposed to DR in combination with either 100 μM or 50 μM afatinib. *n* = 32–40. Statistical significance was tested by one-way ANOVA and the Tukey test. Lifespan significance was tested by the log-rank (Mantel-Cox) test. Significances are marked with lines or corresponding color. ns = not significant, ^*^ = *p* < 0.05, ^**^ = *p* < 0.01, ^***^ = *p* < 0.001, ^****^ = *p* < 0.0001. Scale bar: 100 μm.

Since DR and afatinib showed beneficial (but different) effects on animals with *Egfr^CA^* overexpression, we investigated if the combination of DR and afatinib produced superior effects on cellular phenotypes. We compared all previous results with results from combination intervention experiments (Figure 4). Quantification of esg+ cells showed that DR and afatinib reduced cell numbers to levels seen in *w^1118^* control animals; this reduction is significantly greater than the effect of either treatment alone (Figure 4A). Combination treatment did not reduce the number of mitotically active stem cells below the number observed with afatinib treatment alone, but the number of mitotically active cells equaled that of the *w^1118^* controls (Figure 4B). Further, we quantified the cell turnover by counting the RFP+ cell progeny. We found that combination treatment significantly reduced the number of RFP+ nuclei compared with either DR or afatinib, showing that the combination treatment had a superior effect (Figure 4C). The reduced number of GFP-positive cells and their RFP+ progeny were clearly visible in microscopic images (Figure 4D). Transverse cuts showed that a combination of DR and afatinib was able to combine the effects of both interventions by reinstating the epithelial monolayer and reducing number and size of esg+ cells in the gut (Supplementary Figure 1). When we measured the luciferase activity in whole animals, which is indicative for the amount of esg+ cells, the combination treatment produced similar effects to each single treatment, which were below the activity measured in *w^1118^* controls (Figure 4E). In *w^1118^* controls, DR combined with 100 μM afatinib reduced luminescence to a similar level to that produced by DR alone (Supplementary Figure 3). We also quantified the effect of the combination treatment on lifespan. We observed that combination treatment resulted in a similar lifespan to that seen in animals subjected to DR alone and in untreated *w^1118^* controls (Figure 4F). The combination treatment prolonged the median lifespan to 103% (31 d), which was longer than the median lifespan that resulted from DR alone (90%, 27 d) (Table 1, Figure 4G).

This result prompted us to investigate if DR also has a positive effect in combination with a lower afatinib concentration. DR combined with 50 μM afatinib resulted in the same median lifespan as that produced by DR alone (93%, 28 d). Application of 50 μM afatinib alone only marginally increased lifespan (Figure 1F, Table 1).

### DR, afatinib, and combination application in a real-life treatment scenario

**Figure 5:**
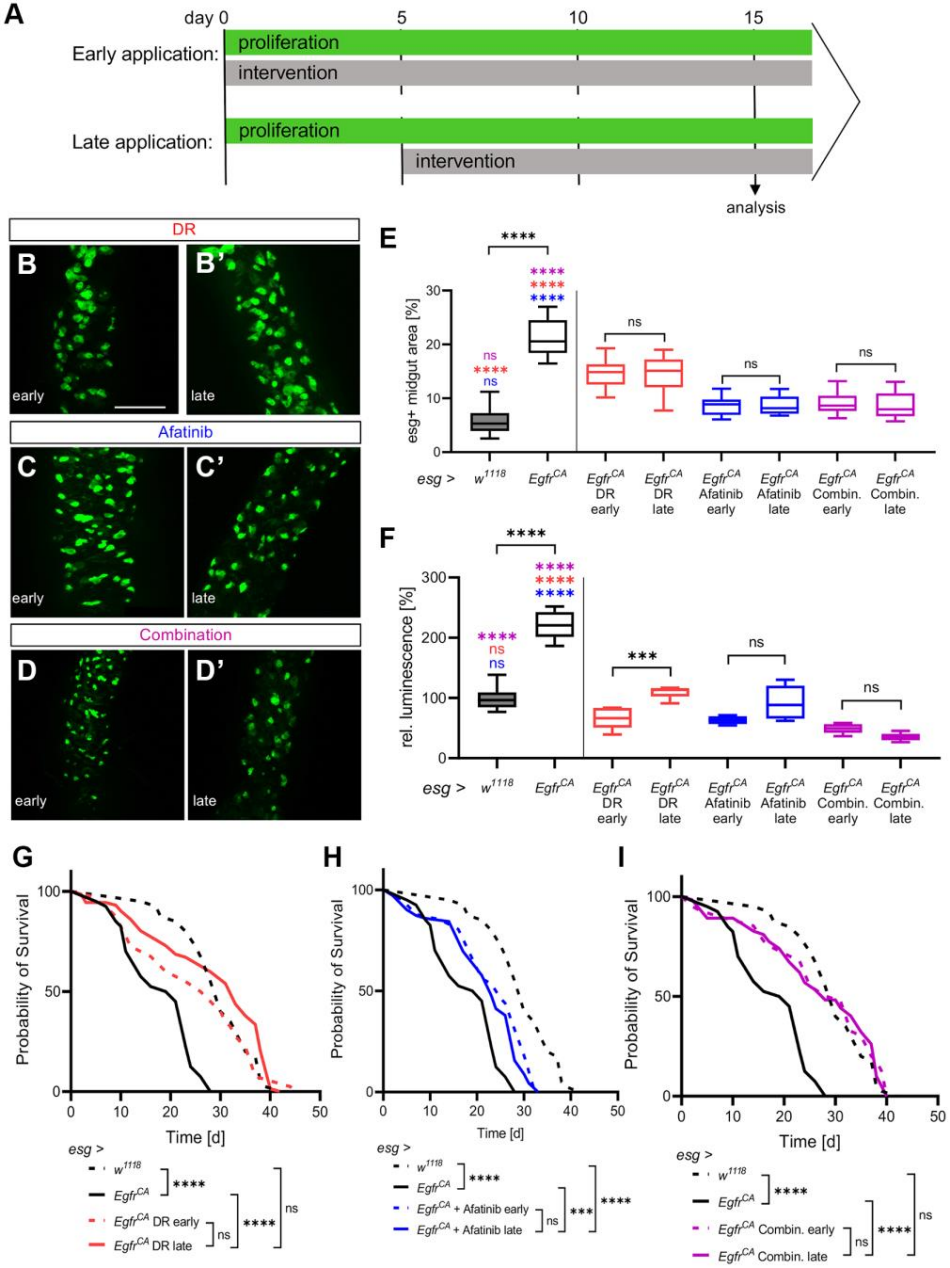
**Effects of DR and afatinib on established phenotypes.** (**A**) Control animals (*esg > w^1118^*) and animals with *Egfr*-induced over-proliferation (*esg > Egfr^CA^*) in intestinal stem cells and enteroblasts (esg+ cells) were given interventions (100 μM afatinib, DR, or a combination of afatinib and DR) either immediately for 15 days (early application, upper row) or 5 days after the onset of over-proliferation for 10 consecutive days (late application, lower row). (**B**–**D**) Anterior midguts of animals exposed immediately (early application) to DR, afatinib, or the combination of DR and afatinib. (**B’**–**D’**) Anterior midguts of animals exposed to DR, afatinib, or a combination of DR and afatinib for 10 days after the onset of over-proliferation to simulate a real-life situation. (**E**) Quantification of the area covered by GFP-positive cells indicating the number and phenotype of esg+ cells in the midgut. *n* = 10–12. (**F**) Luciferase and GFP were expressed simultaneously and quantified in whole animals to measure the over-proliferation phenotype. *n* = 5–6. (**G**–**I**) The lifespan of animals exposed to an early application or late application. *n* = 32–69. Statistical significance was tested by one-way ANOVA and the Tukey test. The lifespan significance was tested by the log-rank (Mantel-Cox) test. Significances are marked with lines or corresponding color. ns = not significant, ^***^ = *p* < 0.001, ^****^ = *p* < 0.0001. Scale bar: 100 μm.

The results presented until now are based on the innervations being applied from the point of induction of the over-proliferation onwards. This model measures an early intervention from tumor initiation on, rather than effects on an established tumor. To create a scenario that is more comparable to CRC treatment in patients, we applied the interventions to an established tumor phenotype. We now refer to these two paradigms as early and late application (Figure 5A). Animals with *Egfr^CA^* expression were first induced for 5 days to reach an advanced tumor phenotype. Quantification of the phenotype and microscopy were performed after 10 consecutive days of treatment. The *Egfr^CA^* animals were exposed to DR, treated with afatinib, or exposed to a combination of both; DR and afatinib. We compared the results of these experiments with results obtained from the *w^1118^* control, the untreated *Egfr^CA^* animals and the early application on day 15 (Figure 5B–5F). Firstly, we quantified the area covered by GFP-positive cells and esg+ cells in the midgut. There was no difference between the late application and the early application for DR, afatinib, or the combination of DR and afatinib (Figure 5E). Similar observations were obtained from microscopic images (Figure 5B–5D, 5B’–5D’). The effects of afatinib alone or afatinib in combination with DR (but not DR alone) were similar to the effects seen in untreated *w^1118^* controls. The approach with late application was used to quantify the number of esg+ cells in the whole animal (as measured by luciferase activity) and these results were compared with those obtained from the early application (Figure 5F). There was no difference between the early and the late application in animals treated with afatinib or afatinib combined with DR. By contrast, DR alone was more effective in reducing the number of esg+ cells in an early situation. The combination of DR and afatinib in a late application reduced the number of esg+ cells to a level below the number observed in *w^1118^* controls (Figure 5F). Furthermore, we tested if treatment with DR, afatinib, or a combination of DR and afatinib prolonged the lifespan of animals overexpressing *Egfr^CA^*. Similar to results obtained in the experiments with early application, each intervention slightly modulated the median lifespan but there was no significant difference between the early and late application. The slight differences observed in median and maximum lifespan are possibly related to the reduced duration of the treatment regime (Figure 5G–5I, Table 1). Thus, the positive effects of DR and afatinib are evident not only upon immediate application, but also with later treatment that more closely resembles the situation that occurs in cancer patients.

## DISCUSSION

Cancer is sensitive to dietary interventions that limit the supply of nutrients [43]. A protein-restricted diet and fasting are associated with a lower cancer risk [8, 44–46]. Despite these findings, DR is yet to gain acceptance in clinical practice, which can be attributed to various ambiguities and practical implementation problems. For this reason, we analysed the effects of a well-defined protein reduction diet in an intestinal tumor model and compared the effects of this DR with those of a targeted pharmacological treatment. We found that the combination of a specific pharmacological intervention and protein restriction completely rescued all relevant tumor-associated phenotypes. We performed the project with the fruit fly *Drosophila* because it offers many advantages; besides the unchallenged wealth of genetic tools, it allows lifespan to be used as the most relevant read-out for both pharmacological and dietary interventions. The effects of DR and CR are well-established in *Drosophila*, especially the lifespan- and healthspan-prolonging effects [47]. Although the lifespan-extending effects of protein restriction are not fully understood, effects on insulin signaling [48] and TOR signaling [47] might not only explain these effects, but also the beneficial effects on tumor growth. Our experiments confirmed that a protein-restricted diet prolongs lifespan. Dietary protein restriction was sufficient to restore the lifespan of animals overexpressing *Egfr^CA^* to that seen in control animals. Interestingly, the effects of dietary protein restriction on cellular proliferation were mild. Our findings are in line with other studies showing that a protein-restricted diet reduces tumor growth in human and mouse xenograft models by modulating the IGF/mTOR pathway [9, 12]. Beyond this, a protein-restricted diet enhances the effects of immunotherapy and causes reprogramming of tumor-associated macrophages [49]. Protein restriction improves insulin and leptin sensitivity in patients with prostate cancer [50]. To avoid complete protein reduction in the diet, some studies depleted intake of dietary methionine, which reduces the growth of KRAS-driven human CRC xenografts in mice both as a preventive and a treatment approach [51]. In a mouse mammary cancer model, methionine reduction decreases proliferation and increases apoptosis [52]. Interestingly, we found no evidence that DR induces a wasting phenotype, both under control conditions and in the cancer model. It remains elusive if this lack of indicators of cachexia is due to our model representing a relatively mild type of intestinal tumors or if this is of general relevance. This has to be taken into account when applied in clinical settings. In obese patients with prostate cancer, restriction of protein intake reduces body mass; however, this outcome was intentional [50]. The depletion of dietary methionine intake during chemotherapy does not affect body mass index and bodyweight in cancer patients [53, 54]. If DR does induce a cachectic phenotype, then a recently developed recurrent DR regimen might be helpful, as it induces the positive effects of DR, while enabling animals to receive a control diet for more than 50% of the experimental time [48].

One highly interesting result of the current study was the observation that protein restriction combined with a specific pharmacological agent rescued all relevant phenotypes of the cancer model, namely, the over-proliferation of intestinal cells and the reduced lifespan. Targeted cancer therapy using highly specific inhibitors is not likely to capture all the relevant components of the complex network of interacting signaling pathways involved in cancer. As such, targeted therapy will probably not rescue all possible disease-associated phenotypes. We used the *Egfr^CA^* over-proliferation model to mimic EGFR alterations observed in human CRC and treated this model with the specific EGFR inhibitor afatinib. Afatinib had beneficial effects on the over-proliferation phenotype in the intestine but was largely ineffective in altering lifespan. Afatinib is an effective standard treatment for NSCLC associated with activating *EGFR* mutations [55], and it has shown potential in a *Drosophila* lung tumor model associated with *Egfr^CA^* expression [41]. EGFR inhibition is a recommended targeted treatment strategy in CRC [56]. In the current study, afatinib was used as a prototype pharmacological intervention that specifically targets the relevant oncogene. It did not rescue all relevant phenotypes. The high doses of targeted therapies that are needed to obtain their full effectiveness at EGFR might be associated with the development of severe side effects that impair lifespan. Inhibiting several pathways or molecules through combinatorial treatment could be an effective approach for treating complex disease phenotypes. Combinatorial approaches can show additive or even synergistic effects by targeting several proteins in a cancer-signaling network. Except for a study in which methionine restriction was combined with chemotherapy [57], the effectiveness of a protein-restricted diet in combination with a targeted therapy has not been studied sufficiently. By combining afatinib with dietary protein restriction, we brought together the lifespan-prolonging effect of DR with the pharmacologically induced decrease in number of mitotically active cells. This regime could serve as a blueprint for incorporating nutritional interventions into treatment approaches. In addition, the combination of pharmacological and dietary interventions restored the cell turnover rate to that seen in healthy conditions and thereby reduced the growth more efficiently than either afatinib or DR alone. It seems that the protein-restricted diet intervenes in pathways that reduce cell growth and cell division that are not inhibited by afatinib (that is, not the EGFR/MAPK pathway). A possible target for DR is insulin signaling. Combining IGF-1R inhibition with EGFR inhibition turned out to be a possibility in using the EGFR inhibitors gefitinib and osimertinib but not erlotinib [58–60]. These results suggest that combining an EGFR inhibitor with an intervention that reduces insulin signaling is a promising strategy for cancer therapy. Afatinib combined with DR did not alter lifespan to a greater extent than either treatment alone, but with focus on the median lifespan the combination of both turned out to be more effective. An advantage of combining a pharmacological inhibitor with DR is that the toxicity of the drug can be minimized. An increased tolerance to radiotherapy or chemotherapy induced by fasting, energy restriction, or methionine intake restriction prior to the therapy has been repeatedly shown [54, 61–66]. Moreover, a fasting-induced sensitization to radio- and chemotherapy (which has been shown *in vitro* and *in vivo*) might account for these positive effects of an additional and accompanying DR [67–70]. In addition, fasting or fasting-mimicking diets enhance the effectivity of combinations of MAPK inhibitors [13, 14].

Taken together our results show that DR, and more precisely a protein-restricted diet, rescues relevant disease phenotypes in a mild intestinal cancer model. Most importantly, DR normalizes lifespan. The full potential of DR can be seen when DR is used in combination with therapeutic strategies, such as EGFR inhibitors, where all relevant tumor-associated phenotypes are rescued. Thus, the inclusion of different forms of DR into cancer treatment strategies is a promising approach that may improve patient outcomes.

## CONCLUSIONS

In the frame of this manuscript, we were able to show that a reduced amount of protein in the ingested diet has a significant positive effect on EGFR-induced intestinal tumors. We used a *Drosophila* model in which we could precisely adjust the amount of protein in the diet. Protein restriction was shown to have a particularly positive effect on restoring normal life expectancy. In combination with the specific EGFR inhibitor afatinib, all major tumor-associated phenotypes could be normalized. These effects could also be demonstrated in a real treatment scenario where interventions were started after the full tumor phenotype was expressed. The results presented here clearly demonstrate that dietary restriction, in particular the reduction of protein in the diet, shows a positive effect on tumor diseases of the intestine and that, consequently, such intervention strategies should be meaningfully incorporated into therapy regimens.

## MATERIALS AND METHODS

### Fly lines and husbandry

The following fly stocks were used in this study: *esg-Gal4, mCD8::GFP/CyO; H2B::RFP, tub-Gal80ts/TM6* (gift from Tobias Reiff, Düsseldorf), *+/+; p{Esg-gal4}, p{UAS-GFP}, p{tubulin-Gal80ts}/p{Esg-gal4}, p{UAS-GFP}, p{tubulin-Gal80ts}; p{UAS-Luciferase at attp2}/p{UAS-Luciferase at attp2}* [32], *w[1118]* (Bloomington Stock Center, #5905), w[^*^]; *P{w[+mC] = Egfr.2.A887T.UAS}8-2* (Bloomington Stock Center, #9533).

Flies were raised on cornmeal–molasses *Drosophila* medium at room temperature. Flies were transferred to holidic media for experiments. Afatinib, dissolved in DMSO, was diluted in EtOH before applied to media. Control media were treated with DMSO (0.1%). Media for use with afatinib were prepared with low-melt agarose instead of agar-agar. 2 ml was aliquoted into each 28 ml *Drosophila* culture vial.

### Media

A chemically defined holidic diet was used for DR experiments, which was modified after Piper et al. [40]. Restriction of dietary protein intake was attained by reducing the amino acid content of the diet so that the amino acid:carbohydrate ratio was 1:16.

### Luciferase quantification

The luciferase assay was performed using the ONE-Glo luciferase assay system (Promega). Flies were exposed to induction at 29°C for 5 or 15 days. Three adult flies per replicate were transferred into 150 μL Glo lysis buffer (Promega, #E2661) and homogenized in a bead homogenizer (OMNI bead ruptor 24, OMNI International) at 3.25 m/s for 2 min. After centrifugation at 3000 × g for 3 min, 50 μl of the sample was mixed with 50 μl of ONE-Glo substrate (Promega, #E6110). Luciferase luminescence was measured with a plate reader (Synergy H1 Plate Reader, Biotek Instruments Inc.,).

### Immunohistochemistry and microscopy

Midguts of flies were dissected in 1x PBS post-induction at 29°C. Flies that were subjected to the ReDDM system and tissues that were used for cell area measurements were submitted directly to fluorescence microscopy. DNA was stained with DAPI (Roth GmbH, RotiMount Flour-Care DAPI #HP20.1). Flies that were used in immunohistochemistry experiments were induced for 10 days at 29°C. The midguts were fixated with 4% paraformaldehyde for 45 min. The midguts were washed for 3 x 10 min in 0.1 PBST (PBS + 0.1% Triton X-100 (Roth GmbH, #3051.2) and incubated for 1 h with blocking buffer (0.1% PBST + 5% normal goat serum). Primary antibodies (Cell Signaling technology Inc, pH3 Histone H3 Rabbit AB #4499; Developmental Studies Hybridoma Bank, DHSB-GFP-8H11-S Mouse AB) were added (1:150 in blocking buffer) and incubated overnight at 4°C. The midguts were washed 3 × 10 min with 0.1% PBST. Secondary antibodies (Jackson Immuno Research Inc., Alexa Flour 488-conjugated AffiniPure Goat α-Mouse IgG #115-545-205, Cy3-conjugated AffiniPure Goat α-Rabbit IgG #111-165-003) were added (1:300 in blocking buffer) and incubated at 4°C overnight. Midguts were washed 3 × 10 min with 0.1% PBST and DAPI (1:2000 in 0.1% PBST; DAPI #6843.3, Roth GmbH) was added for 10 min. Tissues were washed once for 10 min in 0.1% PBT and mounted on glass slides in 80% glycerol. Microscopy was performed using a Zeiss Axio imager Z.1 with an apotome and the AxioVision software (Axiovision SE64 Rel.4.9).

### Coupled colorimetric assay

Body fat was quantified using the method of Hildebrandt et al. [71]. Samples for body fat quantification were collected after 5 and 15 days of consecutive induction at 29°C. Five flies per replicate were collected in screw-cap tubes and the fly weight was assessed. Next, 1 ml of 0.05% PBST was added before samples were homogenized with a bead homogenizer (OMNI bead ruptor 24, OMNI International) for 2 min at 3.25 m/s. Samples of homogenated flies were heated for 5 min at 70°C to inactivate enzymatic activity. Samples were centrifuged for 3 min at 3000 × g to separate fly debris and supernatant. To measure body fat, a triglyceride standard was prepared by adding 1 μl trioleate (Sigma-Aldrich, #T7140) to 455 μl 0.05% PBST. The triglyceride standard was homogenized and heat-inactivated using the same conditions used for sample preparation. The standard was 2-fold diluted with 0.05% PBST from 100 μg/50 μl to 3.25 μg/50 μl. All samples were heated to 37°C in a heat block and centrifuged for 3 min at 2500 × g. Then, 50 μl of each sample or standard was pipetted onto a transparent 96-well microtiter plate. The absorbance at 500 nm was measured with a microplate reader (Synergy H1 plate reader, BioTek Instruments Inc.) at T0. Next, 200 μl of triglyceride reagent solution (Pointe Scientific, #T7532500) was added to each well, and the plate was incubated for 30 min at 37°C with mild shaking (200 rpm). The absorbance was measured at 500 nm to obtain T1 values. The triglyceride equivalent content per fly was calculated using a standard curve.

### Bicinchoninic acid assay

Quantification of body protein content was performed after 5 and 15 days of consecutive induction at 29°C by using the same samples used in the coupled calorimetric assay. The protein content of flies was measured with a bicinchoninic acid assay (BCA) using a microplate assay according to the manufacturer’s protocol (Pierce BCA protein assay kit #23227, Thermo Fisher Scientific). For each replicate, 25 μl of the sample was pipetted into a transparent 96-well plate, and 200 μl of freshly mixed working reagent was added to each well. The plate was shaken for 30 sec to mix the reagents. After incubation at 37°C for 30 min, the absorbance was measured with a microplate reader (Synergy H1 plate reader, BioTek Instruments Inc.) at 562 nm.

### Analysis of esg+ cell area and RFP-positive nuclei

Flies were induced for 5 consecutive days at 29°C before midguts were dissected. For analysis of esg+ cell area, female F1 progeny of crossings with the *esg/GAL4-UAS/GFP-UAS/Luciferase* line were used. To quantify RFP-positive nuclei, female F1 progeny of the esg-GAL4 ReDDM line were used. The images for both analyses were obtained with fluorescence microscopy. To exclude observer bias in the quantification of esg+ regions of the midgut, randomly selected (2–3 per replicate) rectangles with an area of 100 μm^2^ were placed over different midgut regions. For this purpose, only the DAPI channel was used so that no information about the esg+ signal of this midgut region is available to the observer. For analysis of esg+ area only, the GFP channel of the squares and rectangles were exported as an 8-bit image for analysis in Image J (version 1.49). To count RFP-positive nuclei, only the DsRed channel of the squares or rectangles was exported. Nuclei were counted with Image J.

### Lifespan analysis

Flies were age-matched and mated. Per replicate, a group of ten female flies was kept on 2 ml of holidic diet in 28 ml vials; vials were maintained at 29°C. Flies were counted three times per week until all flies had died. Media and vials were changed twice per week minimum.

### Vibratome sectioning

Flies used for vibratome sectioning were kept on 29°C for 5 consecutive days upon the respective media and treatment. Flies were decapitated and legs and wings were removed. Posterior abdomen was partly removed with scissors. Thorax and remaining abdomen were pricked with pointed minutien pins. Then animals were submerged in 4% PFA for > 12 h at 4°C for tissue fixation. Fixated animals were then embedded into 7.5% agarose. Agarose cubes containing flies were cut in slices with a vibratome (Hyrax V 50, Zeiss) through the transverse plane at a frequency of 80 Hz, 0.5 mm amplitude and 100 μm thickness. Slices were incubated with GFP-Booster (1:200 in blocking buffer) overnight at 4°C. Then slices were washed with 0.1% PBST for 10 min before being stained with Phalloidin (1:1000 in buffer; Flash Phalloidin™ Red 594 #424203, BioLegend) and DAPI (1:200 in blocking buffer; DAPI #6843.3, Roth GmbH) for 10 min. Next, slices were washed again 3 × 10 min with 0.1% PBST before mounted on glass slides for microscopy.

### Statistics

Graph Pad Prism v.7 was used for statistical analysis. Normality was tested with the D’Agostini and Pearson test, or the Shapiro-Wilke test if the sample size was small. One-way ANOVA and the Tukey test were performed for data that conformed to a gaussian distribution. For datasets that included non-gaussian-distributed data, the Kruskal-Wallis test and Dunn’s test were performed. The significance of the results of the lifespan assay was calculated with the Mantel-Cox-test. Median–maximum lifespan of the oldest 10% of flies per group was calculated using Microsoft Excel (Microsoft Office Professional Plus 2016).

## Supplementary Materials

Supplementary Figures
