# Supplementary material for: Low-protein diet applied as part of combination therapy or stand-alone normalizes lifespan and tumor proliferation in a model of intestinal cancer
Source: Aging (Albany NY). 2021 Nov 12;13(21):24017–36. doi: 10.18632/aging.203692 (PMC8610115; doi:10.18632/aging.203692)
Supplement: Supplementary Figures [file aging-13-203692-s001.pdf]

SUPPLEMENTARY FIGURES

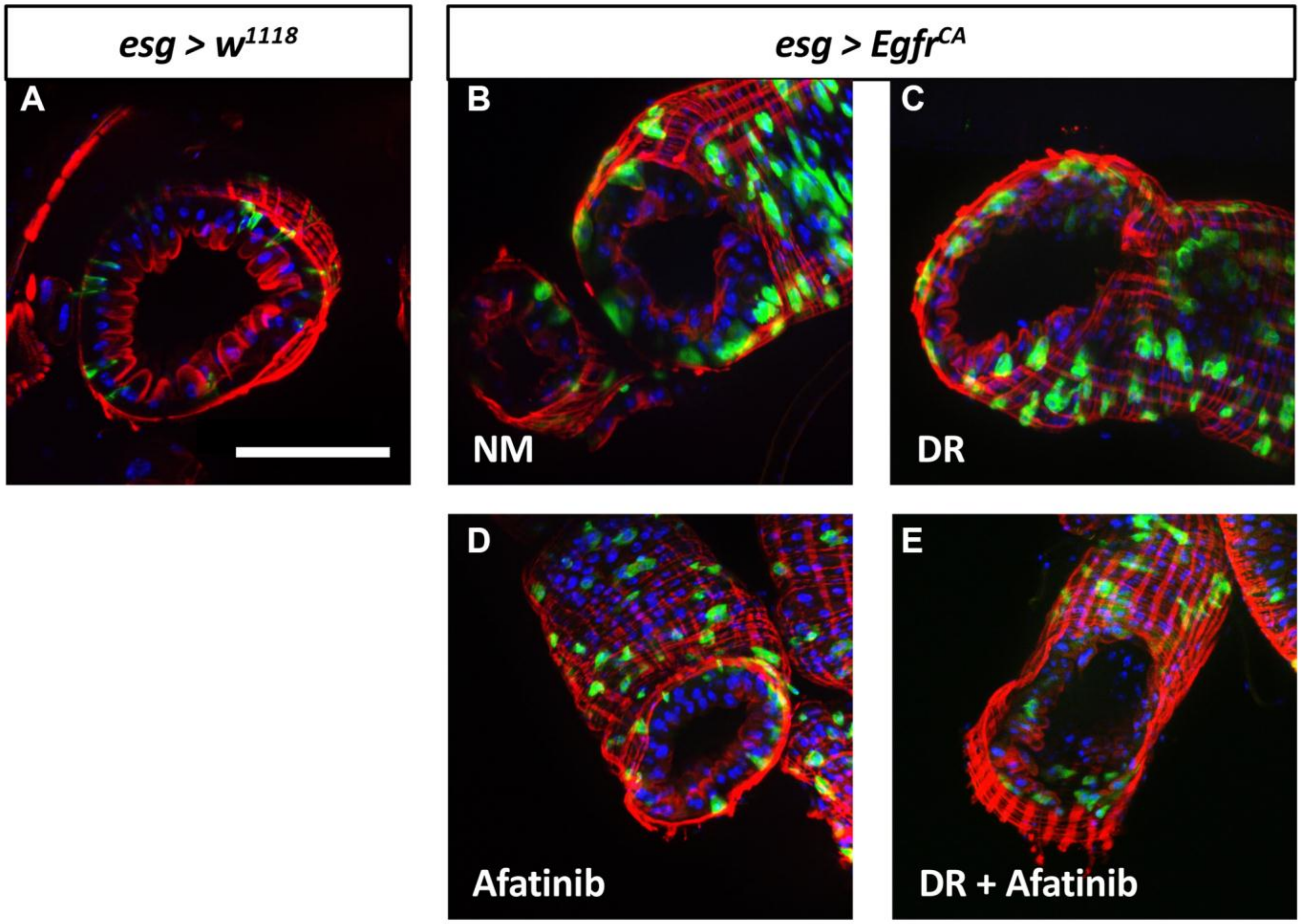

Supplementary Figure 1. Transverse sections through *Drosophila* abdomen of controls (*esg > w^{1118}*) and *$Egfr^{CA}$* (*esg > $Egfr^{CA}$*) after 5 days of induction at 29°C. (**A**) Control animals on NM. (**B**) *$Egfr^{CA}$* animals on NM, (**C**) DR, (**D**) 100 µM Afatinib or (**E**) DR + 100 µM afatinib. Abbreviations: NM = normal medium; DR = dietary restriction; green = GFP: ISC; blue = DAPI: DNA; red = Phalloidin, actin. Scale bar = 100 µm.

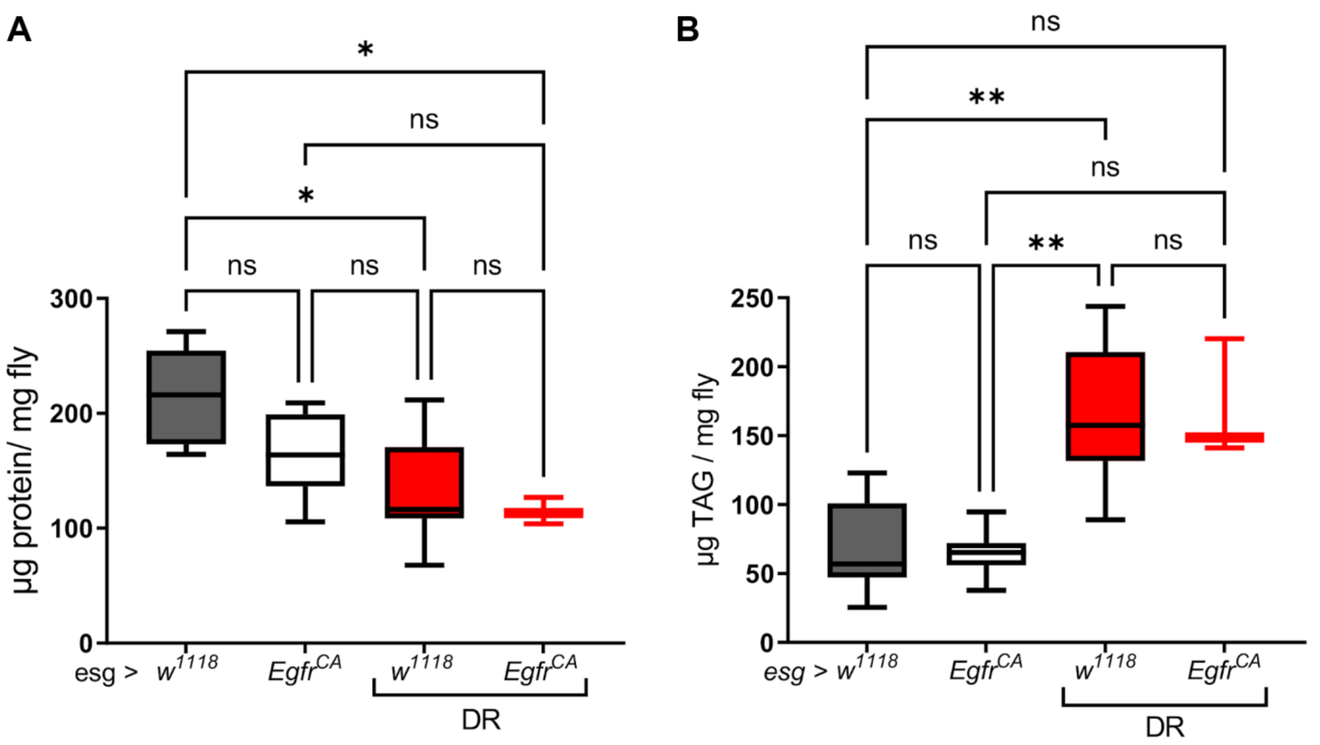

Supplementary Figure 2. (A) Protein content and (B) triacylglyceride content of controls (*esg* > *w*^{1118}) and *esg* > *Egfr*^{CA} after 15 days of induction. Abbreviation: DR = dietary restriction. Significances were calculated with ANOVA and Tukey test and are marked with lines. ns = not significant, * = $p < 0.05$, ** = $p < 0.01$, *** = $p < 0.001$, **** = $p < 0.0001$. n = 3–11.

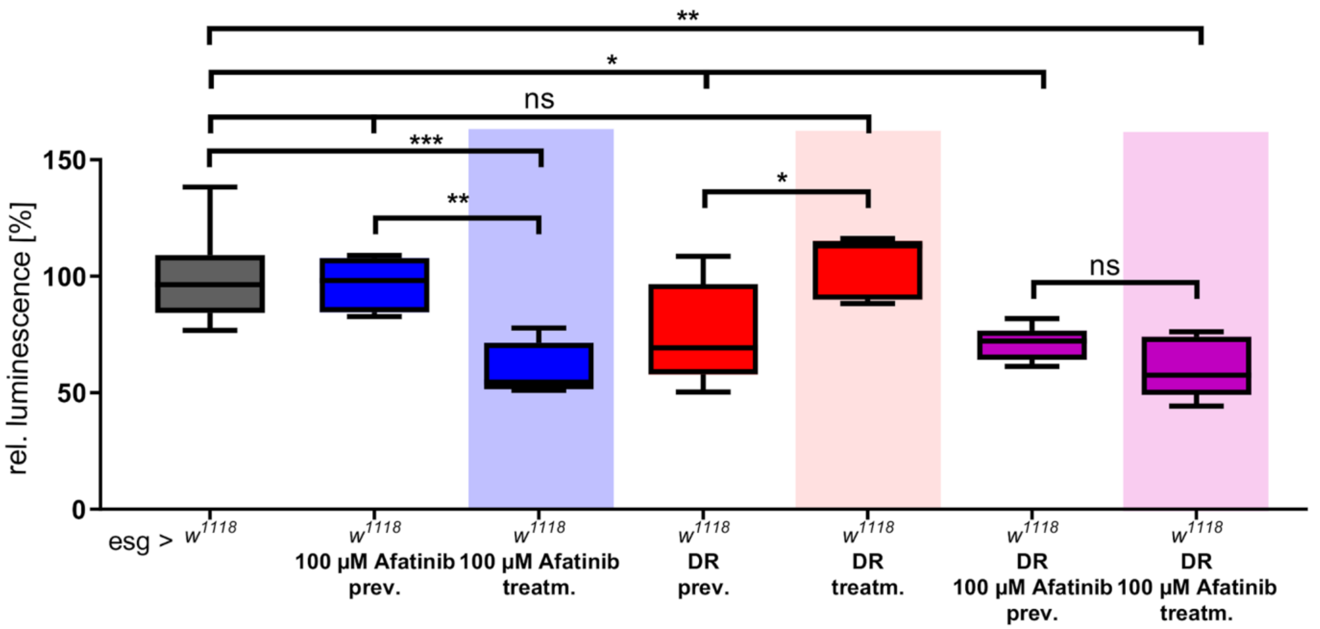

Supplementary Figure 3. Relative luminescence of *esg* > *w*^{1118} animals after 15 days of induction at 29°C exposed to dietary restriction and afatinib. Abbreviations: DR = dietary restriction, prev. = prevention Significances were calculated with one-way ANOVA and Tukey test and are marked with lines. ns = not significant, * = $p < 0.05$, ** = $p < 0.01$, *** = $p < 0.001$, **** = $p < 0.0001$. n = 5–8.
